# Neonatal immune responses to TLR2 stimulation: Influence of maternal atopy on Foxp3 and IL-10 expression

**DOI:** 10.1186/1465-9921-7-40

**Published:** 2006-03-21

**Authors:** Bianca Schaub, Monica Campo, Hongzhen He, David Perkins, Matthew W Gillman, Diane R Gold, Scott Weiss, Ellice Lieberman, Patricia W Finn

**Affiliations:** 1 University Children's Hospital Munich, Department of Pulmonary, LMU, Munich, Germany; 2 Pulmonary and Critical Care Division, Department of Medicine, Brigham and Women's Hospital, Harvard Medical School, Boston, MA, USA; 3 Department of Ambulatory Care and Prevention, Harvard Medical School and Harvard Pilgrim Health Care, Boston, MA, USA; 4 Immunogenetics and Transplantation, Department of Medicine, Brigham and Women's Hospital, Harvard Medical School, Boston, MA, USA; 5 Channing Laboratory, Brigham and Women's Hospital, Harvard Medical School, Boston, MA, USA; 6 Harvard Medical School, Boston, MA, USA

## Abstract

**Background:**

Maternal atopic background and stimulation of the adaptive immune system with allergen interact in the development of allergic disease. Stimulation of the innate immune system through microbial exposure, such as activation of the innate Toll-like-receptor 2 (TLR2), may reduce the development of allergy in childhood. However, little is known about the immunological effects of microbial stimulation on early immune responses and in association with maternal atopy.

**Methods:**

We analyzed immune responses of cord blood mononuclear cells (CBMC) from 50 healthy neonates (31 non-atopic and 19 atopic mothers). Cells were stimulated with the TLR2 agonist peptidoglycan (Ppg) or the allergen house dust mite Dermatophagoides farinae (Derf1), and results compared to unstimulated cells. We analyzed lymphocyte proliferation and cytokine secretion of CBMC. In addition, we assessed gene expression associated with T regulatory cells including the transcription factor Foxp3, the glucocorticoid-induced TNF receptor (GITR), and the cytotoxic lymphocyte antigen 4 (CTLA4). Lymphocyte proliferation was measured by ^3^H-Thymidine uptake, cytokine concentrations determined by ELISA, mRNA expression of T cell markers by real-time RT-PCR.

**Results:**

Ppg stimulation induced primarily IL-10 cytokine production, in addition to IFN-γ, IL-13 and TNF-α secretion. GITR was increased following Ppg stimulation (p = 0.07). Ppg-induced IL-10 production and induction of Foxp3 were higher in CBMC without, than with maternal atopy (p = 0.04, p = 0.049). IL-10 production was highly correlated with increased expression of Foxp3 (r = 0.53, p = 0.001), GITR (r = 0.47, p = 0.004) and CTLA4 (r = 0.49, p = 0.003), independent of maternal atopy.

**Conclusion:**

TLR2 stimulation with Ppg induces IL-10 and genes associated with T regulatory cells, influenced by maternal atopy. Increased IL-10 and Foxp3 induction in CBMC of non-atopic compared to atopic mothers, may indicate an increased capacity to respond to microbial stimuli.

## Background

The early immunological mechanisms that predispose to the development of allergic immune responses are the focus of recent studies [1]. Prior investigations have examined the development of allergen-specific T cell memory cells that is dominated by T helper 2 (Th2) cytokines [2]. The immunological events that drive T helper memory development are initiated early in infancy [3], possibly even in utero [4,5]. Already during the perinatal period there are immunological differences in neonates at high risk of allergy, namely relatively reduced capacity for type 1 (Th1) interferon gamma (IFN-γ) responses, compared with low risk neonates with no family history of allergy [6-8].

Th1 responses such as production of IFN-γ and IL-12 can be influenced by innate, non-antigen-dependent immune stimulation. Innate immune stimulation is in part mediated via mammalian toll-like receptors (TLR). Conserved throughout evolution, TLRs participate in innate immune responses to a variety of microbial pathogens, for example the cell wall component of gram-positive bacteria, peptidoglycan (Ppg), i.e. predominantly recognized by TLR2 [9-13], but specific cellular responses in the early immune system have just started to be a focus of research [14]. While murine models as well as epidemiological studies suggest an involvement of TLR4 agonists in modulating asthma or allergic diseases [15,16], TLR2 agonists can also decrease allergic immune responses in murine models [17]. Recent human data demonstrated increased levels of TLR2 in children of farmers exposed to high microbial burden, where a very low prevalence of atopy occurs [18]. Also, genetic variation in TLR2 was described to be a major determinant of the susceptibility to asthma and allergies in children of farmers [19].

We hypothesized that innate stimulation of cord blood mononuclear cells (CBMC) with a TLR2 agonist might influence T cell responses in cord blood mononuclear cells depending on a maternal background of atopy. Specifically, we tested whether the innate TLR 2 agonist Peptidoglycan (Ppg) influences immune factors in addition to the secretion of Th1 (IFN-γ, IL-12), Th2 (IL-13) cytokines and TNF-α. We examined T cell subsets such as T regulatory cells, which are characterized by secretion of the cytokines IL-10, TGF-β, and expression of e.g. the transcription factor Foxp3 and GITR. As maternal atopy is known to increase the risk for atopic diseases in children, we hypothesized that regulatory factors of T cells may be diminished in CBMC of mothers with atopy.

## Methods

### Human study populations

Fetal cord blood samples (n = 50) were obtained from Boston area pregnancies for laboratory-based analysis. The subjects for the study were recruited during the prenatal period to participate in one of two ongoing pregnancy studies [20,21]. Umbilical cord blood was obtained at the time of delivery from healthy neonates born at term after uncomplicated pregnancies. The laboratory investigators were blinded to clinical information, and samples were analyzed based on sample availability to perform the laboratory studies.

At the time of enrollment all mothers completed a questionnaire regarding atopic status. Maternal atopy was determined by detailed interview or questionnaire during pregnancy and was defined as a history of doctor's diagnosis of asthma and/or hay fever, and/or eczema. Of the 50 cord blood samples analyzed, unblinding revealed that 31 of the mothers had no maternal history of atopy, and 19 mothers had maternal atopy. Of these 19, 2 mothers had a doctor's diagnosis of asthma, 2 of them had also asthma and hay fever; 7 mothers had only hay fever, 4 had only eczema, and 4 mothers had a doctor's diagnosis of hay fever and eczema. Demographic data regarding maternal age and smoking, delivery type, offspring gender, birth weight and ethnicity were not significantly different between the two groups of atopic and non-atopic mothers. Specifically, there were no smokers in any of the groups. Exclusion criteria included multiple gestation (twins, triplets), and inability to answer questions in English. Informed consent was obtained from mothers for their participation in the study, including cord blood collection. Approval was obtained from the human research committee of the Brigham and Women's Hospital and Harvard Pilgrim Health Care, Boston, MA.

### Isolation of CBMC and lymphocyte proliferation

Cord blood samples were collected from the umbilical vein after delivery and processed fresh, non cryo-preserved as previously described [22,23]. Samples were placed in heparinized tubes and processed within 24 hours. Cord blood mononuclear cells (CBMC) were isolated by density-gradient centrifugation with Ficoll-Hypaque Plus (Pharmacia, Uppsala, Sweden) after dilution in phosphate buffer saline (PBS, Sigma Aldrich, St.Louis, MO). Cells were washed in RPMI 1640 and diluted in 10% human serum (Biowhittaker, Walkersville, MD) to a concentration of 5 × 10^6 ^cells/ml. For the lymphocyte proliferation assay 0.5 × 10^6 ^cells/well were cultured in triplicates in 96-well round-bottom tissue-culture plates (Corning, NY, NY) for 3 days, stimulated with peptidoglycan (Ppg, 10 μg/ml, Staph. Aureus, Sigma Aldrich, St. Louis, MO), Dermatophagoides farinae (Derf1, 30 μg/ml, Indoor Biotechnologies, Charlottesville, VA), or phytohemagglutinin (PHA, 5 μg/ml, Sigma Aldrich, St.Louis, MO) as positive control and compared to unstimulated samples. The positive control PHA induced CBMC proliferation with a stimulation index (SI) of 33 ± SEM 8. The doses for anti-MHC II and anti-CD4 (each 10 μg/ml, BD Bioscience Pharmingen, San Jose, CA) and corresponding isotype controls and for the previous stimuli were established in prior dose and time-course experiments. Specificity of Ppg for TLR2 was determined in prior experiments using TLR2 -/- mice demonstrating lack of spleen cell proliferation after Ppg stimulation. As control, Ppg stimulation in TLR4 -/- mice demonstrated increased lymphocyte proliferation of spleen cells. Endotoxin concentrations in Ppg, Derf1, and PHA preparations, measured by Limulus assay, were very low (<0.01 EU/ml = 0.002 ng/ml), and did not significantly change lymphocyte proliferation or cytokine secretion in CBMC. By testing the functional ability of CBMC with different doses of LPS and active components such as Lipid A, LpA (starting at 0.01 ng to 100 ng/ml), we detected increased lymphocyte proliferation with doses of LpA above 1 ng/ml; therefore levels below 0.01 ng/ml had no influence on the analysis.

After incubation, samples were pulsed with 1 μCi ^3^H-Thymidine for an additional 8 hours. Cultures were performed at 37°C in a humidified 5% CO_2 _incubation chamber. Cells were harvested with a Tomcat Mach II harvester (Wallac, Turku, Finland) onto filter plates, which were read using a β-Counter. Proliferation was either assessed by counts per minute (cpm) or quantified by stimulation index (SI), which is calculated as the ratio of mean counts per minute (cpm) of stimulated over unstimulated replicates.

### Cytokine measurements

Cells cultured in media were harvested immediately and cell cultures, stimulated with Ppg, Derf1 or PHA as described above, were harvested after 3 days of stimulation. Supernatants were aliquoted in duplicate into 96-well plates (50 μl/well), which are precoated with cytokine specific antibody. Optical density was measured at 450 nm. The cytokines IL-13, IFN-γ, IL-12, TNF-α and IL-10 were measured by ELISA (Endogen, Rockford, IL) according to the manufacturer's instructions. The sensitivity of the assay was 7 pg/ml for IL-13, 2 pg/ml for IFN-γ, 3 pg/ml for IL-12 (p70), 5 pg/ml for TNF-α and 3 pg/ml for IL-10.

### Real-time quantitative RT-PCR

For RNA, CBMC were stimulated with the described stimuli for 3 days at 5 × 10^6 ^cells/ml in 6-well plates. Total RNA was isolated from CBMC with TRI Reagent (Sigma-Aldrich, St. Louis, MO). Isolated RNA was reverse transcribed with SuperScript II RNAse reverse transcriptase (Life Technologies, Carlsbad, CA). Specific primer pairs for GAPDH and β-actin (housekeeping genes), TLR2, Foxp3, GITR, CTLA4 and TGF-β were designed with the Primer Express software (Applied Biosystems, Foster City, CA). The sequences of the forward (FW) and reverse (RE) primer pairs used in the experiments were as follows: GAPDH: TTGTGGAAGGGCTCATGACC (FW), TCTTCTGGGTGGCAGTGATG (RE), β-actin: CTATTGGCAACGAGCGGTTC (FW), AGGAAGGCTGGAAAAGAGCCT (RE), TLR2: CATTCCCTCAGGGCTCACAG (FW), TTGTTGGACAGGTCAAGGCTT (RE), Foxp3: GAGAAGCTGAGTGCCATGCA (FW), GGTCAGTGCCATTTTCCCAG (RE), GITR: CGAGGAGTGCTGTTCCGAGT (FW), TGGAATTCAGGCTGGACACAC (RE), CTLA4: ATC GCC AGC TTT GTG TGT GA (FW), GACCTCAGTGGCTTTGCCTG (RE); TGF-β: TTCAACACATCAGAGCTCCGA (FW), GGAGAGCAACACGGGTTCAG (RE). Direct detection of the PCR product was monitored by measuring the increase in fluorescence caused by the binding of SYBR Green to dsDNA. Using 5 μl of cDNA, 5 μl of primer, and 10 μl of SYBR Green Master Mix (Applied Biosystems) per well, the gene-specific PCR products were measured continuously by means of GeneAmp 5700 Sequence Detection System (Applied Biosystems) during 40 cycles. All experiments were run in duplicate, and the same thermal cycling parameters were used. Non-template controls and dissociation curves were used to detect primer-dimer conformation and non-specific amplification. The threshold cycle (C_T_) of each target product was determined and set in relation to the amplification plot of GAPDH. The C_T _is the number of PCR cycles required for the fluorescence signal to exceed the detection threshold value. The detection threshold was set to the log linear range of the amplification curve and kept constant (0.3) for all data analysis. The difference in C_T _values of two genes was used to calculate the fold difference. The level of mRNA of the individual gene is described as gene expression. The relative quantitative results were used to determine changes in gene expression in stimulated as compared to unstimulated samples [24,25].

### Statistical analysis

Data analysis was performed with SigmaStat software. Data for lymphocyte proliferation, cytokine concentrations and gene expression were not normally distributed and could regularly not be transformed to normality. Non-detectable cytokine concentrations were assigned to a value of 0.01 for inclusion into the analysis. Non-parametric tests (Kruskal-Wallis, Mann-Whitney) were used to compare the median of cytokine levels, proliferation values or gene expression between different groups. Statistically significant differences for the comparison of several groups were determined by one-way ANOVA analysis followed by a comparison of groups with the Tukey-Kramer analysis. Data are either reported as mean ± SEM or median ± CI depending on the distribution and presented as box- and whiskers- plots (Median, whiskers: 5% and 95%-quantile) with outliers. We used either Pearson's or Spearman's correlation to assess the association between cytokine secretion and gene expression. Statistical significance was defined by p < 0.05.

## Results

### Stimulation of CBMC with innate and adaptive stimuli

In CBMC of healthy neonates, we detected constitutive expression of TLR2. TLR2 expression assessed by real time RT-PCR was increased 3.46 fold (± 1.5) following stimulation with the TLR2 agonist Ppg as compared to unstimulated cells. TLR2 was also expressed on the cell surface of mononuclear cells following Ppg stimulation detected by flow cytometry (not shown).

**Figure 1:**
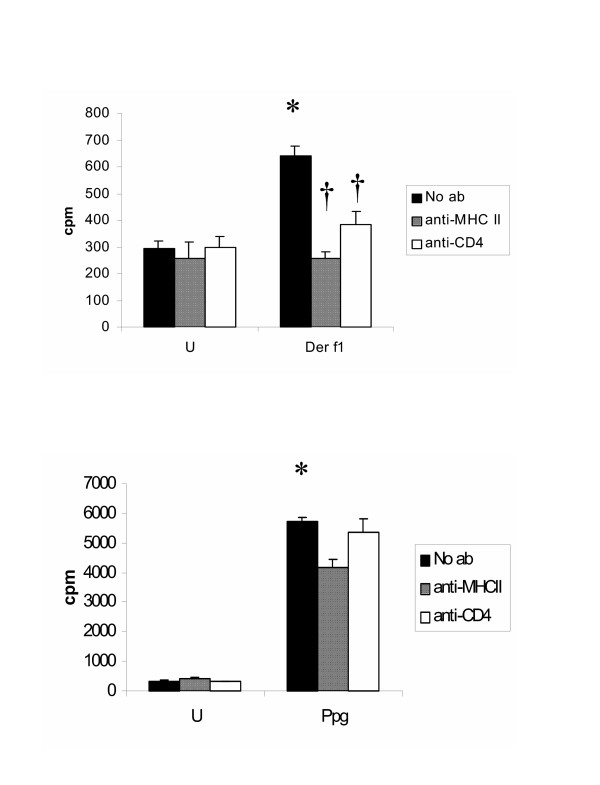
**A+B. **Lymphocyte proliferation following addition of anti-MHC II or anti-CD4 ab is unchanged in unstimulated CBMC and following stimulation with the innate stimulus Ppg. Following addition of anti-MHC II or anti-CD4 ab, lymphocyte proliferation is decreased after stimulation with the allergen Derf1 (p < 0.05). **A+B. **Lymphocyte proliferation is shown in counts per minute (cpm) and was determined after stimulation with the indicated dose of Ppg and Derf1 (30 μg/ml) for 72 h by ^3^H-Thymidine uptake as described in Methods (n = 50). Anti-MHC II or anti-CD4 ab was applied in a dose of 10 μg/ml each.

Allergic (house dust mite Der f1) and innate, non-allergic stimulation (Ppg) of CBMC led to significantly increased proliferation following stimulation (Fig. 1A, B, black bar, no antibody) as compared to unstimulated cells (U). Allergen-induced lymphoproliferation was shown to be specific for the allergen Der f1 through blockade of lymphoproliferation by either anti-MHCII or anti-CD4 antibodies (Fig. 1A). Also, we present data demonstrating, as expected, that the innate stimuli Ppg is not inhibited by addition of anti-MHCII or anti-CD4 antibodies (Fig. 1B).

### Regulation of cytokine secretion through innate stimulation

**Figure 2:**
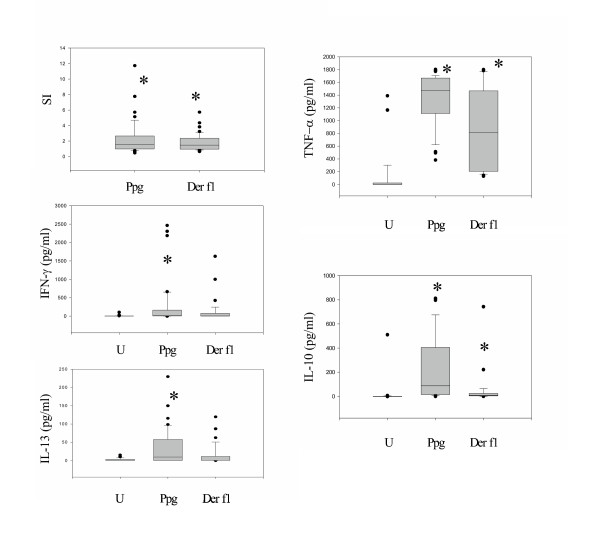
**A. **Lymphocyte proliferation following stimulation with Ppg and Derf1 was increased in CBMC (p < 0.001). **B. **IFN-γ secretion was increased following stimulation with Ppg as compared to unstimulated CBMC (U) (p < 0.001). **C**. IL-13 secretion was increased following Ppg stimulation as compared to unstimulated cells (U)(p = 0.001). **D**. TNF-α production was increased following stimulation with either Ppg or Der f 1 compared to U (p < 0.001). **E. **IL-10 production was increased following stimulation with Ppg as compared to U (p < 0.001). **A-E. **Lymphocyte proliferation and cytokine concentrations from supernatants of CBMC were determined following stimulation with the indicated doses of Ppg and Derf1. Lymphocyte proliferation shown as SI (stimulation index, ratio of mean counts per minute of stimulated over unstimulated replicates) was measured by ^3^H-Thymidine uptake, cytokine concentrations were measured with ELISA (Methods)(n = 50). Data are shown as Box- and whiskers- plots (Median, whiskers: 5% and 95%-quantile) with outliers.

To analyze the effect of innate stimuli on effector cell responses in CBMC, we determined lymphoproliferative responses and Th1 (IFN-γ, IL-12 (p70)) and Th2 (IL-13) cytokine production as well as production of the pro-inflammatory cytokine TNF-α and the immunoregulatory cytokine IL-10. Lymphoproliferation was increased following Ppg stimulation compared to unstimulated cells (p < 0.05), and higher as compared to Der f1-induced proliferation (Fig. 2A). IFN-γ secretion was increased following stimulation with Ppg as compared to unstimulated cells (p < 0.001) (Fig. 2B). There was mildly increased IL-12 (p70) secretion, though at low levels and not significant (p = 0.18, data not shown). IL-13 was significantly elevated following Ppg stimulation as compared to unstimulated cells (p = 0.001) and also increased, though not significantly, after Der f1 stimulation (p = 0.18). TNF-α production was significantly increased following innate (Ppg) and allergic stimulation (both p < 0.001). IL-10 secretion was significantly increased following stimulation with Ppg as compared to unstimulated cells (p < 0.001), and higher than following Derf1 stimulation.

### Influence of maternal atopy on cytokine secretion

We have previously shown that allergen-induced (OVA) proliferation in CBMC from mothers with a diagnosis of asthma was increased as compared to mothers without asthma [26]. Here, we determined whether maternal atopy has an influence on lymphoproliferation and cytokine responses to innate and allergic stimulation. Lymphoproliferative responses and Th1 (IFN-γ, IL-12) as well as Th2 (IL-13) cytokine responses to innate and allergic stimuli were comparable in CBMC with and without maternal atopy (Table 1, data not shown). For all cytokines, median concentrations in unstimulated CBMC were low. TNF-α secretion as a representative pro-inflammatory cytokine was very high following innate stimulation and similar in mothers with and without atopy. Interestingly, IL-10 secretion was significantly higher following Ppg stimulation in CBMC without maternal atopy as compared to CBMC with maternal atopy (p = 0.03, Table 1).

**Table 1:** Association of maternal atopy* with decreased IL-10 production following innate stimulation (Ppg) in CBMC.

**Parameter**	**Stimulus**	**No maternal atopy (Median, 25/75%)**	**Maternal atopy (Median, 25/75%)**	**P value (Mann-Whitney rank)**
**SI**	Ppg	1.67 (0.99/2.41)	1.45 (1.13/2.75)	0.97
	Der f1	1.43 (0.98/2.35)	1.38 (0.93/1.95)	0.93

**IFN-γ**	U	2.49 (1.05/5.83)	2.89 (1.86/4.37)	0.57
	Ppg	7.53 (4.70/136.20)	6.07 (2.71/19.19)	0.22
	D	3.70 (1.25/22.38)	9.48 (2.46/18.60)	0.73
**IL-13**	U	0.01 (0.01/3.39)	0.01 (0.01/3.69)	0.58
	Ppg	8.04 (0.01/46.04)	6.50 (0.01/61.04)	0.91
	D	0.01 (0.01/16.78)	4.10 (0.01/16.96)	0.64

**TNF-α**	U	2.25 (0.01/176.85)	0.27 (0.01/7.60)	0.17
	Ppg	1474.00 (1295.50/1645.00)	1529.00 (1069.12/1694.25)	0.87
	D	758.30 (205.25/1340.25)	782.50 (168.30/1470.00)	0.96

**IL-10**	U	1.01 (0.52/2.24)	0.38 (0.01/0.77)	0.007
	**Ppg**	**69.54 **(25.92/239.78)	**13.47 **(9.12/86.22)	**0.03**
	D	10.28 (4.15/14.92)	6.51 (4.25/13.80)	0.68

* Maternal atopy was defined as history of doctors diagnosis of one or more of the diagnoses asthma, hay fever or eczema. N = 31 mothers without and n = 19 mothers with atopy (differs slightly in groups depending on availability of data).

Lymphocyte proliferation is shown as SI (stimulation index, ratio of mean counts per minute of stimulated over unstimulated replicates). Cytokine concentrations were measured with ELISA and are presented in pg/ml.

### T cell subpopulations

**Figure 3:**
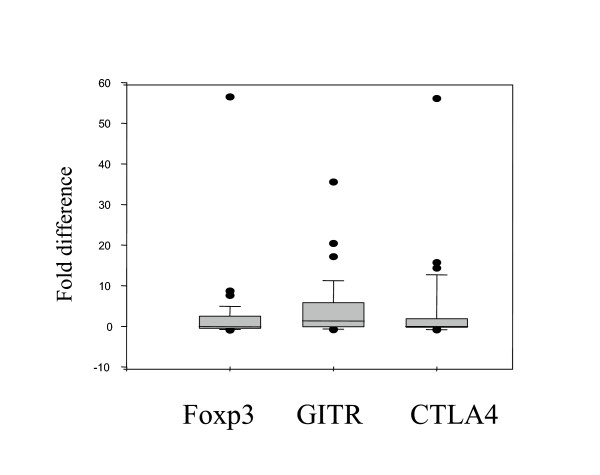
Gene expression of GITR following stimulation with Ppg was increased as compared to unstimulated cells (p = 0.07). The mRNA level of the individual gene is shown as fold difference in gene expression in Ppg (10 μg/ml) stimulated as compared to unstimulated samples and compared to the housekeeping gene GAPDH. RNA was prepared as described in Methods (n = 50). Quantitative gene expression was assessed with real-time RT-PCR. Data are shown as Box- and whiskers- plots (Median, whiskers: 5% and 95%-quantile) with outliers.

IL-10 is secreted from several cell types including macrophages and characteristically produced from a subpopulation of T cells with regulatory capacity (T regs). As T cells express TLR2 and Ppg stimulates proliferation, we determined important markers of these T cell subsets by real-time RT-PCR such as expression of the transcription factor Foxp3, the glucocorticoid-induced TNF receptor GITR, the cytotoxic lymphocyte antigen 4 CTLA4 and the cytokine TGF-β on CBMC. GITR expression was increased following stimulation with Ppg as compared to unstimulated cells, though not significantly (p = 0.07)(Fig. 3). Foxp3 and CTLA4 were both constitutively expressed at low levels (data not shown), and increased following stimulation with Ppg, however not significantly (Fig. 3). TGF-β was expressed at low levels at baseline and decreased after stimulation with Ppg as compared to unstimulated cells (p = 0.03)(data not shown). Stimulation with the allergen Der f1 resulted in non-significant changes in expression of Foxp3, CTLA4 and GITR (not shown). To investigate this population of T cells further, we assessed the percentage of CD4^+^CD25^+ ^cells, one predominant phenotype of T regulatory cells following stimulation with Ppg. We found a mild, non-significant increase in the percentage of CD4^+^CD25^+ ^cells after stimulation with Ppg (data not shown).

### Effect of maternal atopy on markers of T cell subpopulations

To determine the importance of maternal atopy on parameters of subsets of T cells in addition to IL-10, we assessed the expression of Foxp3, GITR and CTLA4 depending on maternal atopy (Table 2). Following stimulation with Ppg, differences in T cell markers in CBMC from children of mothers without as compared to those with maternal atopy became apparent. Foxp3 and CTLA4 were both increased in CBMC of children of mothers without as compared to those with maternal atopy; the differences were marginally significant for Foxp3 (p = 0.049)(p = 0.17 for CTLA4). As IL-10 and Foxp3 were significantly higher in CBMC from children of mothers without atopy, we further assessed the correlation between IL-10 and Foxp3. Foxp3 was positively correlated with IL-10 secretion in CBMC following stimulation with Ppg (r = 0.53, p = 0.001, Table 3). These positive correlations were seen in CBMC from both children of mothers without and with maternal atopy (r = 0.52, p = 0.01 and r = 0.56, p = 0.06, data not shown). Also, positive correlations were demonstrated for increased Ppg-induced IL-10 secretion with GITR (r = 0.47, p = 0.004) and CTLA4 (r = 0.49, p = 0.003), independent of maternal atopy.

**Table 2:** Association of maternal atopy* with decreased Foxp3 expression following Ppg stimulation in CBMC.

**Parameter**	**No maternal atopy (Median, 25/75)**	**Maternal atopy (Median, 25/75)**	**P value (Mann-Whitney rank)**
**Foxp3**	**1.59 (**-0.4/3.26)	**-0.6 **(-0.78/1.06)	**0.049**
**GITR**	1.22 (-0.17/4.28)	2.74 (-0.40/7.21)	0.43
**CTLA4**	1.19 (-0.22/2.02)	- 0.13 (-0.77/0.79)	0.17

* Maternal atopy was defined as history of doctors diagnosis of one or more of the diagnoses asthma, hay fever or eczema. N = 31 mothers without and n = 19 mothers with atopy (differs slightly in groups depending on availability of data).

The mRNA level of the genes is shown as fold difference in gene expression in stimulated as compared to unstimulated samples and compared to the housekeeping gene GAPDH. Quantitative gene expression was assessed with real-time RT-PCR.

**Table 3:** Correlation between IL-10 production and specific markers of T regulatory cells in the whole population (n = 50, differs slightly in groups depending on availability of data).

	**Correlation Coefficient r**	**p †**
**TGF-β **	0.02	0.95
**Foxp3**	**0.53**	**0.001**
**GITR**	**0.47**	**0.004**
**CTLA4**	**0.49**	**0.003**

† Spearman rank test

## Discussion

This study demonstrates that microbial stimulation with the TLR2 agonist peptidoglycan in vitro modulates functional immune capacities of cord blood mononuclear cells (CBMC) from children of mothers with as compared to without a doctors diagnosis of maternal atopy. In CBMC from children of mothers without a doctors diagnosis of atopy, an increase of Ppg-induced IL-10 secretion was paralleled by an increase of two markers of T regulatory cells (significantly for Foxp3 and mildly for CTLA4). In addition, Ppg stimulation was associated with a positive correlation between IL-10 and genes associated with T regulatory cells (Foxp3, GITR and CTLA4), suggesting innate modulation of T regulatory cells in CBMC. These data support the hypothesis that microbial stimulation of CBMC leads to immune modulation in association with the maternal atopic background.

Of note, the phenotype of T regulatory cells is not clearly defined to date. We acknowledge the limitation of a mixed CBMC population in this study. While this study did not address cell type, prior studies indicate that TLRs are present not just on monocytes and B cells but also on T cells, underscoring a putative link between innate and adaptive immunity [32]. The induction of both IL-10 and IFN-γ following stimulation with Ppg in this study could indicate a role of a specific population of T cells in human CBMC. For example, it has been proposed that IL-10 and IFN-γ producing CD4^+ ^T cells may be one of the human equivalents of the CD4^+^CD25^+ ^T regulatory cells originally described in the mouse [33]. In addition, in this study not only IL-10 but also GITR, another marker characteristic for T regulatory cells, was increased following Ppg stimulation. We present an increase of TLR2-stimulated IL-10 as well as a correlation between IL-10 and other markers of T regulatory cells. These data may indicate that microbial stimulation such as Ppg can impact T cells in the fetal immune system, potentially capable of regulating several immune processes including cytokine secretion. This is intriguing in the context that Ppg stimulation in our murine model of asthma could decrease allergic stimulation [17].

IL-10 secretion may be crucial in modulating the development of the fetal immune system, and in contributing to Th2 maturation via inhibition of IL-12 production [27]. On the other hand, regarding allergic diseases, IL-10 was demonstrated in several studies to be associated with lower risk for atopy or sensitization to egg protein in later life [28,29]. The Ppg-induced increase of IL-10 in our study could indicate a role for innate stimuli in early immunomodulation. Furthermore, IL-10 was induced in chronic schistosomiasis in African children, who have a low prevalence of atopic disease [30]. Additionally, successful allergen-desensitization therapy has been postulated to work through the induction of IL-10 secreting T regulatory cells. In support of this concept, IL-10 secreting T regulatory cells were shown to be induced by glucocorticoids and β 2-agonists, the hallmark of anti-allergic therapy [31].

Furthermore, the forkhead-winged-helix family transcription factor Foxp3 may control genes encoding T regulatory cell-associated molecules (such as CD25, CTLA4 and GITR). Mutations in Foxp3 lead to the X-linked immunodeficiency syndrome IPEX in humans (immune dysregulation, polyendocrinopathy, enteropathy, X-linked syndrome). Clinical features are autoimmune disease, inflammatory bowel disease, severe allergy including atopic dermatitis, food allergy, and fatal infection [34]. Foxp3 is stably expressed in mature natural T regulatory cells; the role of Foxp3 in the development of the neonatal immune system remains to be determined. It is intriguing that both IL-10 and Foxp3 levels are decreased in cord blood of neonates of mothers with atopy in our study.

Maternal atopy is known to be an important influential factor in a child's allergic predisposition [35]. In this study, maternal atopy is defined as doctors diagnosis of asthma, hay fever and/or eczema. Unfortunately, data on maternal sensitization were not available, which we acknowledge as a potential limitation of the study. From the literature, the prevalence of a positive skin prick test to at least one allergen is reported in up to 60% in the 20–29 year old age range in the American NHANES population not stratified as high or low risk for atopy [36], which most closely represents the population in our study. The percentage of sensitization can therefore be much higher without having ever any atopic symptoms. Also, some studies suggest that a history of atopic symptoms may be more indicative of allergic disease than skin test positivity to allergens.

Our analysis was performed in a group of 50 mothers including 19 with maternal atopy as defined by the doctor's diagnoses asthma and/or hay fever and/or atopic eczema. Further separate analysis in the subgroups were not statistically feasible. In addition, the diagnosis of maternal atopy comprises a common immunological basis for all three diseases. Regardless, the specific immunological mechanisms by which maternal atopy may influence the development of atopy in the child remain undefined. Thus, differences in T cell regulation, possibly T regulatory cells, depending on the maternal atopic background, may be biologically important. The study of Amoudruz et al. in CBMC of 9 mothers with and 10 without allergy is consistent with this concept [14]. In this study, cytokine secretion of IL-6 is lower after Ppg stimulation in CBMC of mothers with as compared to mothers without allergy. Importantly, Pasare et al have shown that the suppressive effects of CD25^+ ^regulatory cells can be blocked by the presence of IL-6, produced by DC and activated through stimulation of the TLR pathways [37]. Our study suggests that in maternal atopy, T regulatory cells may be potentially less effective as demonstrated by reduced secretion of IL-10 and by diminished expression of Foxp3.

## Conclusion

In conclusion, our study provides evidence that exposure to microbial stimuli may induce the neonatal immune system to increase IL-10 secretion. Gene expression related to regulatory T cell subpopulations appears to be influenced by innate stimuli, which may potentially result in an altered phenotype or function of T cell subpopulations. Our findings that IL-10 and Foxp3 expression were reduced in mothers with atopy raise the possibility that CBMC from their neonates may have a diminished capacity to respond to microbial stimuli. Whether these patterns in the context of additional genetic and environmental factors are associated with an increased risk of atopy in the child remains to be investigated.

## Abbreviations

CBMC, cord blood mononuclear cells; LpA, Lipid A; Ppg, Peptidoglycan; TLR, Toll-like receptor.

## Competing interests

The author(s) declare that they have no competing interests.

## Authors' contributions

BS designed the experiments, carried them out, analyzed the results and drafted the manuscript. MC and HH carried out part of the experiments. DP participated in study design and data analysis. MWG, DG, SW and EL contributed to study design, and draft of the manuscript. PWF participated in study design, experimental design, analysis and draft of the manuscript. All authors read and approved the final manuscript.
